# Reduced HIV transmission at subsequent pregnancy in a resource-poor setting

**DOI:** 10.1258/td.2011.100458

**Published:** 2011-07

**Authors:** Felicity Zvanyadza Gumbo, Gwendoline Quintoline Kandawasvika, Kerina Duri, Munyaradzi Paul Mapingure, Nyaradzai Edith Kurewa, Kusum Nathoo, Simbarashe Rusakaniko, Mike Zvavahera Chirenje, Babill Stray-Pedersen

**Affiliations:** * Department of Paediatrics and Child Health; † Department of Immunology; ‡ Department of Community Medicine; § Department of Obstetrics and Gynaecology, University of Zimbabwe, Harare, Zimbabwe; ** Division of Obstetrics and Gynaecology, Rikshospitalet and University of Oslo, Norway

## Abstract

Several studies indicate that HIV-infected women continue to have children. We set out to determine the trend in HIV transmission at subsequent pregnancies. From 2002–2003, pregnant women were enrolled in a single dose nevirapine-based Prevention of Mother-to-Child Transmission of HIV (PMTCT) programme. Six years later, women with subsequent children in this cohort were identified and their children's HIV status determined. From 330 identified HIV-infected mothers, 73 had second/subsequent children with HIV results. Of these, nine (12.3%, 95% confidence interval [CI]: 4.6–20.1%) children were HIV-infected. Of the 73 second children, 51 had older siblings who had been initially enrolled in the study with definitive HIV results with an infection rate of 17/51 (33.3%, 95% CI: 19.9–46.7). About 35% of the women had been on antiretroviral drugs. These results demonstrate lower subsequent HIV transmission rates in women on a national PMTCT programme in a resource-poor setting with the advent of antiretroviral therapy.

## Introduction

With the improvements in HIV care and management, HIV-infected women are now considering having more children. Several reports indicate that women are going through many cycles of PMTCT (Prevention of Mother to Child Transmission of HIV).^1,2^ This problem has become significant in areas of high HIV prevalence, particularly where resources are few and breastfeeding is the norm.

Single dose nevirapine, which has been the mainstay of PMTCT in developing countries, can have an impact on subsequent pregnancies. Its use results in the selection of resistant mutants among HIV-infected women.^3–5^ The effectiveness of a single dose of nevirapine in subsequent pregnancies has been questioned. However, three studies in Africa found that there was no reduction in efficacy when used consecutively.^6–8^ With better access to antiretroviral treatment for pregnant women and better counselling, but with advanced maternal disease, the transmission of HIV infection in subsequent pregnancies in a breastfeeding population makes it important to be sure.

With this background we set out to determine HIV transmission rates among a cohort of mothers who had been enrolled in a PMTCT programme and who had two consecutive pregnancies. We compared known risk factors between the two pregnancies.

## Methods

### Setting

The study was conducted at three primary maternal child health clinics in peri-urban areas around Harare (namely, Epworth, Seke North and St Mary's) in Zimbabwe.

### Design

This was a descriptive cross-sectional study done on a ceased observational cohort of HIV-infected women who had been enrolled in a PMTCT programme and who had been followed up for 15 months after delivery.

### Background

Between 2002 and 2003, pregnant women were enrolled into the Better Health for the African Mother and Child (BHAMC) study from 36 weeks of gestation, after obtaining their informed consent. The aim of the initial study was to explore the role of sexually transmitted infections (STIs) in pregnancy outcome without considering prematurity – a late enrolment was, therefore, ideal. Pre- and post-HIV test counselling was offered as part of the national PMTCT programme. Baseline characteristics collected included: sociodemographic information; medical history of STIs; gynaecological examination findings; specimen collection for full blood counts; serology for herpes simplex type 1 and syphilis; and high vaginal swabs for culture. All HIV-infected women were given a single dose of nevirapine at delivery; their infants had similar doses, according to the national guidelines at that time (HIVNET 012).^9^ The infants were then followed up and the HIV status was determined. The proportion of HIV-infected infants was 21.8% (95% confidence interval [CI] −17.8–25.8) which has been previously reported.^10^

### Study activities

A subsequent pregnancy/second pregnancy was defined as the first pregnancy occurring after the initial enrolment pregnancy. Between 2008 and 2009 mothers who had been enrolled in this study and who had subsequent children/second pregnancy were identified through community support groups and clinic registers. All identified women agreed to participate and were interviewed. The information obtained was validated with known information about the women from a previous study. Important additional information included: the outcome of the enrolment pregnancy; number of children since enrolment; and pattern and breastfeeding duration of subsequent children. The maternal health cards were examined for medical records pertaining to CD4 counts and antiretroviral (ARV) therapy. Blood was drawn from the children for HIV testing. All the information from the mothers and children was collected on the initial visit. Infants who were still breastfeeding had blood samples later collected in order to confirm their HIV infection status after the cessation of breastfeeding.

All HIV-infected children were referred for the appropriate care and support. Informed consent was sought from the mothers and the study was approved by the Medical Research Council of Zimbabwe.

After determining the HIV infection rates in subsequent pregnancies, we retrospectively looked for the HIV results and information on enrolment children of same mothers. We compared the HIV transmission rates for the initial enrolment children and subsequent/second children.

### Laboratory methods

The children's blood samples were processed and tested for HIV with DNA polymerase chain reaction (PCR) 1.5 (Roche Diagnostics, Indianapolis, USA), if the children were aged less than 15 months, and rapid HIV antibody tests, Determine (Abbott Diagnostics, Illinois, USA) and Oraquick (Abbott Diagnostics) if they were aged 15 months or older.^11^ If the Determine and Oraquick test results were discordant, the DNA PCR test was used for confirmation.

### Statistical analysis

Data were entered and analysed using Stata version 10 (Texas, USA). A table showing characteristics of HIV-infected women and their infants with subsequent pregnancy was presented. HIV transmission rates and their 95% CIs were calculated for the second and first pregnancies in the same women. The two sample test for proportion was used to compare these transmission rates. The Student's *t*-test was used to compare means and the Pearson chi-square test was used to compare proportions. Univariate odds ratios (ORs) and their 95% CIs for mother-to-child transmission were calculated for known biological factors, such as reported exclusive breastfeeding duration and mother and infant nevirapine intake for the second pregnancy.

## Results

Table 1 describes the characteristics of the study population, i.e. of mothers with subsequent children. The majority reported being married and 30% had changed their spouses after the initial enrolment. The pill was the common method of contraception (47%). Thirty-nine percent of these women had lost the child from the initial pregnancy for which they had initially been enrolled in the study.

**Table 1:** Characteristics of HIV-infected women and their infants with subsequent pregnancy

Characteristics of HIV-infected women and their second infants	Frequency
Mother's age in years (*n* = 76)	
Mean (standard deviation, SD)	30.4 (4.4)
Marital status (*n* = 74)	
Married	56 (75.7%)
Change of spouse since initial enrolment (*n* = 74)	
Yes	22 (29.7%)
Outcome of first child in cohort (*n* = 75)	
Dead	29 (38.7%)
Type of contraception after first enrolment (*n* = 60)	
Pill	28 (46.7%)
Depo Provera	17 (28.3%)
Condom	8 (13.3%)
Intra uterine contraceptive device	1 (1.7%)
Dual protection	6 (10.0%)
Time between deliveries in years (*n* = 71)	
Mean (SD)	3.7 (1.6)
Mode of delivery (*n* = 78)	
Normal vertex	75 (96.2%)
Birth weight in grams (*n* = 74)	
Mean (SD)	3094 (490)
Child sex (*n* = 80)	
Male	42 (52.5%)
Age of second children in months (*n* = 81)	
Mean (SD)	23 (17)

From 434 HIV-infected mothers, 79 (18.2%) had died by 2008 and 25 (5.8%) were unable to be traced. Of the remaining 330 eligible women, 81 (24.5%) had second/subsequent pregnancies. Of these, eight children were not tested for HIV because they had died before the study. Of the 73 children who were tested, nine (12.3%; 95% CI: 4.6–20.1%) were HIV-infected.

Of the 73 second/subsequent children with HIV infection results, 51 (70%) had older siblings who had been initially enrolled in this study with definitive HIV results. Of these older siblings, 17 (33.3%; 95% CI: 19.9–46.7) were HIV-infected. Of the remaining 22 first siblings, 18 died with no ultimate HIV test (cause of death unknown) and four were lost to follow-up.

The difference in HIV transmission rates between the first and second pregnancies was statistically significant (*P* = 0.005).

A comparison of known factors associated with HIV transmission between the initial and subsequent pregnancy resulted in the following observations.

### Breastfeeding

At initial pregnancy, 36 of the 51 (76.2%) were still breastfeeding at nine months. For the subsequent pregnancy the mean duration of breastfeeding was 10.0 months (standard deviation [sd] 7.2) in those infected with HIV and 10.1 months (sd 5.9) in those who were uninfected (OR 1.0 [0.88–1.22]). The mean duration of exclusive breastfeeding in the older siblings was similar to those who were HIV-infected and those who were uninfected (3.1 months [sd 2.8] and 3.0 months [sd 2.5], respectively). At the subsequent pregnancy, HIV-infected children had a similar duration of exclusive breastfeeding periods (4.2 months [sd 2.6] versus 6.2 months [sd 3.3] for uninfected children, OR 0.76 [0.57–1.02]).

### Antiretroviral drugs

None of the mothers from the initial pregnancy were on highly active antiretroviral drugs (HAART). At the subsequent pregnancy, 8.7% of those on HAART transmitted HIV compared to 9.3% who were not (OR 1.08 [0.14–12.82]). At the initial pregnancy 37.5% did not receive single dose nevirapine transmitted versus 36.8% of those who did receive nevirapine (OR 1.12 [0.23–5.34]). For the subsequent pregnancy, the percentages were 22.2 and 10.2, respectively (OR 2.51 [0.43–13.37]; see Table 2).

**Table 2:** Factors associated with HIV transmission at subsequent pregnancy

Factor	HIV-infected children	HIV-uninfected children	Unadjusted odds ratio (95% confidence interval)
Maternal CD4 count	*n* = 4	*n* = 48	
> =200	3 (7.1%)	39 (92.9%)	1
<200	1 (10.0%)	9 (90.0%)	1.44 (0.02–20.43)
Maternal HAART	*n* = 6	*n* = 60	
On treatment	2 (8.7%)	21 (91.3%)	1
Not on treatment	4 (9.3%)	39 (90.7%)	1.08 (0.14–12.82)
Mother and child nevirapine	*n* = 9	*n* = 58	
Yes	5 (10.2%)	44 (89.2%)	1
No	4 (22.2%)	14 (77.8%)	2.51 (0.43–13.37)
Duration of exclusive breastfeeding in months	*n* = 9	*n* = 55	
Mean (SD)	4.1 (2.6)	6.2 (3.3)	0.76 (0.57–1.02)
Duration of breastfeeding in months	*n* = 9	*n* = 55	
Mean (SD)	10.0 (7.2)	10.1 (5.9)	1.0 (0.88–1.12)

HAART, highly active antiretroviral therapy

### Maternal disease

Viral loads and CD4 counts were not done for the women at the initial pregnancy as it was not part of the standard of care at the time. Among the subsequent births 10 mothers who had CD4 counts of less than 200/mL, one (10%) had an HIV-infected child and three (7.1%) of the mothers with CD4 counts above or equal to 200/mL had a subsequent HIV-infected child (OR 1.44 [0.02–20.43]).

## Discussion

In this study population the HIV vertical transmission at a later pregnancy was two-fifths of the prior pregnancy in the same women. This could be attributed to a higher proportion of women on HAART (35%), compared to none on the initial pregnancy, and a longer exclusive breastfeeding period. This implies that they were given better counselling and early diagnosis and treatment of mastitis which are known risk factors of HIV transmission.^10, 12^ It is of note (although not statistically significant) that HIV-infected children at a subsequent pregnancy had shorter exclusive breastfeeding periods – four months versus six months for the HIV-uninfected children – while the mean duration of breastfeeding was similar (10 months).

Another explanation could be that, at the initial pregnancy, the women were seroconverting with high viral loads and, as a result, were more likely to transmit. It is unfortunate that viral loads were not done on these women in this setting.

The women were counselled on dual contraception in order to prevent re-infection with HIV but only a small proportion reported using dual contraception (which included the condom) as their method and the majority were using the pill. It is important to note that the mean time between the deliveries was 3.7 years which could suggest that the pregnancies were planned. Thirty percent of the women in this study had also changed their spouses which could explain their desire to have children. In a study by Olufemi *et al.* which determined the extent of fertility desires in HIV-infected patients, 63.7% of the 147 participants expressed the desire for children even though 50.4% already had two or more children.^13^

This was a small cross sectional study after a ceased cohort study and we were unable to explore other factors associated with HIV transmission. We did not have complete follow up of the first pregnancies which might have influenced our transmission rates. Some of the children died before receiving definitive HIV testing. However, our main point is that the HIV transmission rates at subsequent pregnancies might be lower because of improved PMTCT in our breastfeeding resource-limited setting. This is an important factor when counselling HIV-infected women who want to have more children and we recommend increasing access to maternal HAART from 35% to 100% in our setting.
